# A novel gain-of-function mutation (W818R) of calcium-sensing receptor in a family with autosomal dominant hypocalcemia type 1

**DOI:** 10.3389/fendo.2026.1806677

**Published:** 2026-05-08

**Authors:** Min Fu, Yanxiang Luo, Kexin Xu, Lixin Guo, Qi Pan

**Affiliations:** 1 Department of Endocrinology, Beijing Hospital, National Center for Gerontology; National Clinical Research Center for Gerontology, The Key Laboratory of Geriatrics of NHC; Institute of Geriatric Medicine, Chinese Academy of Medical Sciences, Beijing, China; 2 Department of Endocrinology, Wuhan Third Hospital, Wuhan, China; 3 Peking University Fifth School of Clinical Medicine, Beijing, China; 4 Center for Human Genome Research, Key Laboratory of Molecular Biophysics of the Ministry of Education, College of Life Science and Technology, Huazhong University of Science and Technology, Wuhan, China

**Keywords:** calcium-sensing receptor, mutation, gain-of-function, autosomal dominant hypocalcemia, hypoparathyroidism, hypocalcemia

## Abstract

**Introduction:**

Autosomal dominant hypocalcemia type 1 (ADH1) is a rare inherited disorder caused by gain-of-function mutations in the calcium-sensing receptor (*CASR*) gene, with clinical manifestations ranging from asymptomatic hypocalcemia to recurrent seizures.

**Methods:**

Clinical evaluation and genetic testing were performed. Through activation of the MAPK signaling pathway,cellular functional validation was conducted to confirm the pathogenicity of this variant.

**Results:**

We report a two-generation family in which the proband presented with lifelong recurrent convulsive episodes and was misdiagnosed with epilepsy for decades, resulting in persistent symptoms despite antiepileptic therapy. His son exhibited similar manifestations. A whole-exome sequencing (WES) analysis was identified a previously unreported heterozygous *CASR* mutation, c.2452T>C (p.Trp818Arg). Functional cellular analyses confirmed that it caused a novel gain-of-function *CASR* mutant.

**Conclusion:**

This case highlights the critical role of genetic testing in the diagnosis of ADH1, particularly in patients with recurrent seizures or neonatal hypocalcemia with hyperphosphatemia and normal renal function. Once the diagnosis is established, treatment options for symptomatic patients include calcium supplementation and calcitriol, aiming to maintain serum calcium at the lower end of the reference range and relieve symptoms.

## Introduction

Autosomal-Dominant Hypocalcemia Type 1 (ADH1) is a form of isolated hypoparathyroidism(HP) caused by activating mutations in the calcium-sensing receptor (*CASR*) gene, located on chromosome 3q13.3-q21 (1). This condition accounts for approximately 21% of non-surgical cases of HP (2) and has a population prevalence of 3.9 per 100,000 individual (3). Clinical manifestations include decreased serum calcium levels, mild hypomagnesemia, and elevated urinary calcium excretion (4). Among ADH1 patients, 27% are asymptomatic, 32% exhibit mild to moderate symptoms, and 41% present with severe symptoms. Seizures are the most common clinical feature, occurring in 39% of patients (5). Long-term complications of chronic hypocalcemia and hyperphosphatemia include central nervous system calcifications, nephrocalcinosis, kidney stones, and renal insufficiency (6).

Multiple genes have been reported to be associated with HP. A previous study conducted a whole-exome sequencing (WES) analysis on a family diagnosed with autosomal dominant HP and identified approximately 300 single nucleotide changes as potential candidates for relevant genetic alterations. After filtering the raw data, they narrowed down the candidates to 22 genes that could harbor disease-causing variants (7). Furthermore, a total of 121 distinct *CASR* mutations associated with ADH1 have been reported, with the vast majority being missense substitutions (94.3%), followed by frameshift (3.3%) and insertion mutations (2.4%) (8). Genetic testing is crucial for determining the underlying cause of HP. In this study, we identified a previously unreported *CASR* mutation, c.2452T>C (p.Trp818Arg), in a family with recurrent convulsive episodes through genetic testing, and demonstrated its pathogenicity through cellular functional analyses.

## Materials and methods

### Plasmid construction and site-directed mutagenesis

The human *CASR* expression plasmid (pCMV-*CASR* [human]-Neo; P23923) was obtained from the MiaoLing Plasmid Platform (China). Site-directed mutagenesis was performed to generate the Trp818Arg (W818R) variant using mutation-specific primers designed by Sangon Biotech (Shanghai, China). The primer sequences were as follows:

Trp818Arg-F: CAGCATGCTCATCTTCTTCATCGTCCGGATCTCCTTCATTCCAGCCTATGC
Trp818Arg-R: GCATAGGCTGGAATGAAGGAGATCCGGACGATGAAGAAGATGAGCATGCTG

PCR amplification was carried out using Premix PrimeSTAR HS DNA Polymerase (Takara, R040) according to the manufacturer’s high-fidelity protocol. Following agarose gel purification, the PCR products were treated with DpnI enzyme (Takara, 1235A) to remove methylated parental plasmid DNA and subsequently transformed into competent Escherichia coli cells for plasmid amplification. All constructs were confirmed by Sanger sequencing.

### Cell culture and transient transfection

HEK293T cells were cultured in Dulbecco’s Modified Eagle Medium (DMEM; Gibco, 12800-017) supplemented with 10% fetal bovine serum (Vazyme, F103) and 1% penicillin–streptomycin solution (Proteintech, PR40022) at 37 °C in a humidified incubator containing 5% CO_2_. Cells were routinely tested for mycoplasma contamination.

For functional characterization of the CASR W818R variant, HEK293T cells were transiently transfected with either wild-type *CASR* plasmid or the *CASR* (Trp818Arg) mutant plasmid using ExFect Transfection Reagent (Vazyme, T101-01). At 48 hours after transfection, cells were harvested for total protein extraction, and CASR protein expression was verified by Western blot analysis.

### Western blot analysis

Whole-cell lysates from HEK293T cells transiently expressing wild-type or mutant CASR were subjected to Western blot analysis to detect the CASR protein expression. Briefly, equal amounts of protein were separated by SDS–polyacrylamide gel electrophoresis and transferred onto polyvinylidene difluoride membranes. Membranes were incubated with a primary antibody against CASR (Proteintech, 19125-1-AP), followed by incubation with horseradish peroxidase (HRP)–conjugated secondary antibody (Biosharp, BL003A).

### MAPK activation assay

Activation of the MAPK signaling pathway was assessed by measuring ERK1/2 phosphorylation using Western blot analysis. Forty-eight hours after transient transfection, cells were incubated in calcium-free DMEM (Gibco, 21068028) supplemented with 10% fetal bovine serum (Vazyme, F101) and 1% penicillin–streptomycin for 6–8 hours to minimize basal phosphorylation levels. Cells were then stimulated for 30 minutes with DMEM containing increasing concentrations of CaCl_2_ (0, 0.5, 1, 2, 4, or 8 mM).

Following stimulation, cells were immediately lysed using immunoprecipitation (IP) lysis buffer supplemented with protease and phosphatase inhibitors, and total protein was collected. Western blot analysis was performed using antibodies against phospho-ERK1/2 (Proteintech, 28733-1-AP) and total ERK1/2 (Proteintech, 66192-1-1g). β-Tubulin was used as a loading control. Densitometric analysis was conducted using Image J software, and ERK1/2 phosphorylation was quantified as the ratio of phospho-ERK1/2 to total ERK1/2.

### Statistical analysis

Statistical analyses were performed using GraphPad Prism version 10.1.2 (GraphPad Software, La Jolla, CA, USA). Differences between groups were evaluated using Student’s t test, two-way analysis of variance (ANOVA), and appropriate *post hoc* multiple-comparison tests, as indicated. A P value of less than 0.05 was considered to indicate statistical significance. This study did not involve animal or *in vivo* experiments and therefore did not require institutional ethical approval.

## Results

### Clinical history and biochemical assessment

A 33-year-old male presented with a more than 30-year history of intermittent convulsions. His first episode occurred shortly after birth and was characterized by fever and generalized convulsions involving all four limbs, without associated paralysis, loss of consciousness, trismus, frothing at the mouth, or incontinence. Despite treatment with intravenous calcium at a local hospital, followed by continued oral calcium supplementation, the convulsions remained intermittent. At 3 years of age, a cranial CT scan revealed multifocal calcifications, prompting discontinuation of calcium supplementation and initiation of carbamazepine following a diagnosis of epilepsy. However, serum calcium and phosphate levels were not evaluated and calcium-phosphate metabolism-related disorders were not considered. A follow-up cranial CT scan at 10 years of age showed progression of the calcifications. Despite these interventions, the patient continued to experience episodic limb convulsions, frequently accompanied by numbness. These episodes typically occurred during febrile or fatigued states, lasted 1–3 min, resolved spontaneously, and occurred 2–3 times per year. In adulthood, both the frequency and characteristics of these episodes remained unchanged. The patient also demonstrated developmental delay, impaired cognitive function, and below-average stature and intellectual ability compared to his peers.

Forty-eight days before admission to Beijing Hospital, the patient presented to Beijing Chaoyang District Hospital. Further inquiry into the family history revealed no history of convulsions among the patient’s parents or sister. The serum calcium was 1.51 mmol/L, while sodium, potassium, and chloride levels were within the normal range. Accordingly, under the physician’s guidance, antiepileptic therapy was discontinued, and calcium supplementation was resumed, consisting of oral calcium carbonate D3 and calcitriol.

Forty-three days prior to admission, initial laboratory evaluations were performed at the outpatient clinic of Beijing Hospital, and the results are summarized in **Table 1**. The results revealed severe hypocalcemia, with a serum calcium level of 1.55 mmol per liter, accompanied by hyperphosphatemia and a parathyroid hormone (PTH) level well below the normal range. The ultrasonography of the parathyroid glands showed no abnormalities. Accordingly, the patient, on his own, increased his dosage of calcium carbonate D3 from 1 tablet three times daily to 2 tablets three times daily (totaling 3.6g of elemental calcium per day), without medical supervision. During this period, measurements performed at a local laboratory showed that the patient’s serum total calcium ranged from 1.37 to 1.75 mmol/L(reference range, 2.1–2.8 mmol/L), with ionized calcium levels fluctuating between 0.7 and 0.9 mmol/L(reference range, 1.09–1.35 mmol/L). Subsequently, the patient escalated his calcium carbonate D3 dose to 3 tablets three times daily (5.4g of elemental calcium per day), hypocalcemia remained unresolved. He was subsequently admitted to our hospital for further evaluation and management.

**Table 1:** Laboratory data.

Variable	Reference range, Beijing hospital	Forty-three days before admission	On admission
Sodium(mmol/liter)	137-147	145	146
Potassium(mmol/liter)	3.5-5.3	3.6	3.3
Chloride(mmol/liter)	96-108	100.2	104.8
Carbon dioxide(mmol/liter)	23-29	28.4	24.4
Urea nitrogen(mg/dl)	2.9-8.2		4.83
Creatinine(umol/liter)	59-104	73	70
Glucose(mmol/liter)	3.9-6.1		4.2
Magnesium(mmol/liter)	0.67-1.04	0.76	0.8
Phosphate(mmol/liter)	0.85-1.51	2.13	1.92
Calcium(mmol/liter)	2.03-2.54	1.55	1.74
Corrected calcium(mmol/liter)		1.45	1.72
25-Hydroxyvitamin D(ng/ml)	≥30	23.5	30.5
Procollagen type 1 N-terminal propeptide	9.06-76.24		44.32
β-C-terminal telopeptide of type 1 x-linked collagen(ng/ml)	0-0.584		0.451
Parathyroid hormone(pg/ml)	15-65	8.9	
Aspartate aminotransferase(U/liter)	15-40		13
Alanine aminotransferase(U/liter)	9-50		12
Total bilirubin(mg/dl)	2-21		17.5
Albumin(g/liter)	40-55	45	41
Globulin(g/liter)	20-40	25	20
24 hour urinary calcium(mmol/24h)	2.5-7.5	4.19	5.61
24 hour urinary phosphate (mmol/24h)	12.9-42	10.01	6.65

*To convert blood urea nitrogen values from millimoles per liter (mmol/L) to milligrams per deciliter (mg/dL), divide by 0.357. To convert creatinine values from micromoles per liter (µmol/L) to milligrams per deciliter (mg/dL), divide by 88.4. To convert glucose values from millimoles per liter (mmol/L) to milligrams per deciliter (mg/dL), divide by 0.05551. To convert magnesium values from millimoles per liter (mmol/L) to milligrams per deciliter (mg/dL), divide by 0.4114. To convert phosphate values from millimoles per liter (mmol/L) to milligrams per deciliter (mg/dL), divide by 0.3229. To convert calcium values from millimoles per liter (mmol/L) to milligrams per deciliter (mg/dL), divide by 0.250. To convert 25-hydroxyvitamin D values from nanomoles per liter (nmol/L) to nanograms per milliliter (ng/mL), divide by 2.496. To convert bilirubin values from micromoles per liter (µmol/L) to milligrams per deciliter (mg/dL), divide by 17.1.

†Reference values are influenced by multiple factors, including the patient population and the laboratory assay methods used. The reference ranges used at Beijing Hospital are based on non-pregnant adults without conditions known to affect the test results and therefore may not be applicable to all patients.

The patient’s 22-month-old son, conceived naturally, had episodes of fever-associated seizures, that was characterized by upward deviation of the eyes, a vacant stare, tetanic limbs, and unresponsiveness. Each episode resolved spontaneously within 4–5 min, with two occurrences in total over the course of one year. The child accompanied his father to Beijing for evaluation, where laboratory tests revealed hypocalcemia with subnormal PTH levels. The ultrasonography of the kidneys, cranial CT and ambulatory electroencephalography were unremarkable.

### Clinical management during hospitalization

**Figure 1:**
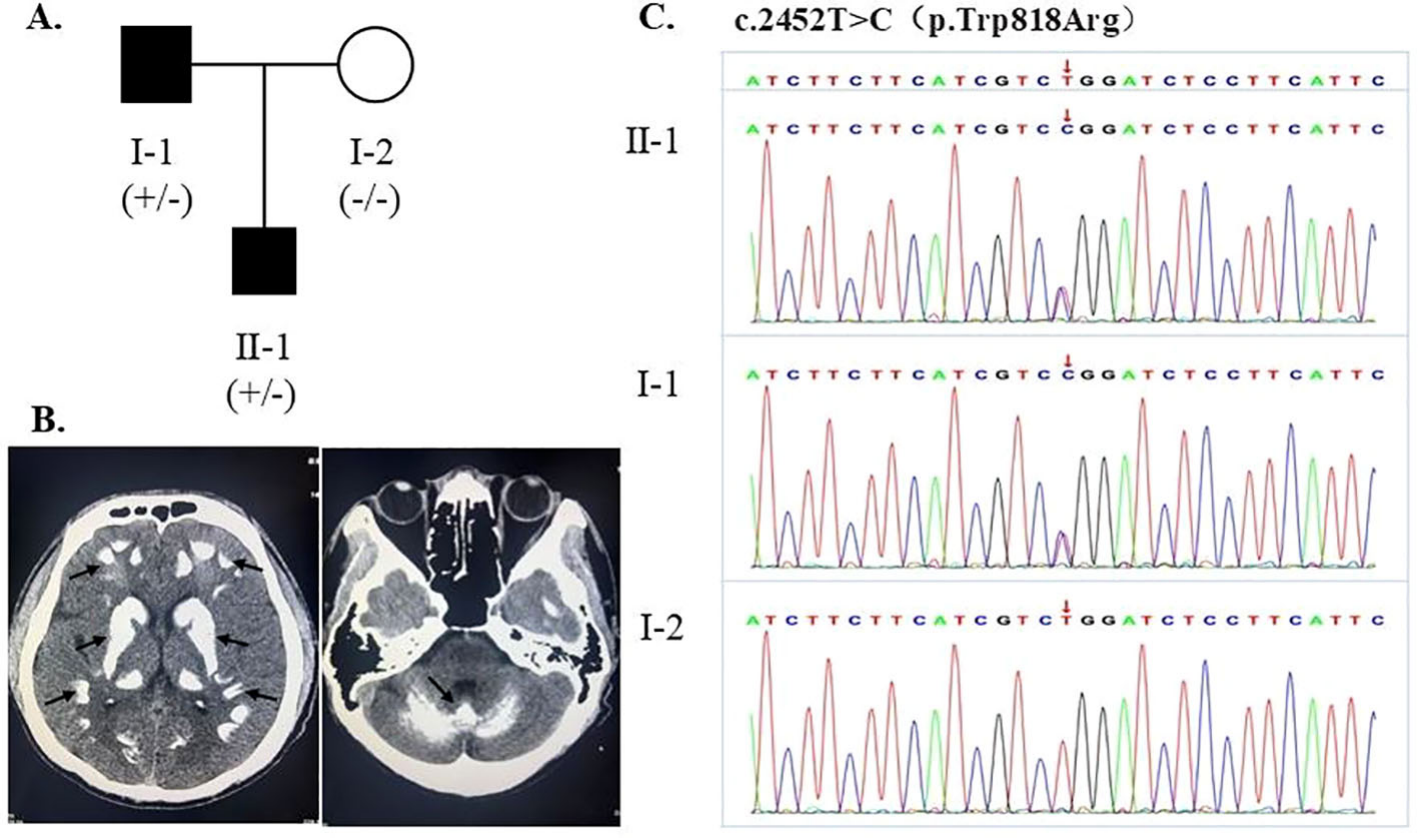
**(A)** Family pedigree. **(B)** Brain CT showing multiple calcifications which are indicated by the black arrow in the bilateral cerebellar hemispheres, thalami, basal ganglia regions, and the corticomedullary junctions of cerebral hemispheres. **(C)** Sanger sequencing showing a *CASR* heterozygous mutation (c.2452T > C) in the proband and his son, and wild-type sequence in his wife.

After admission to Beijing Hospital, a thorough physical examination was performed. The patient’s vital signs were stable. He was 166cm tall and weighed 53.5kg. The skin on the trunk was covered with small, firm, rough papules of a flesh-colored hue. Examination of the spine and joints revealed no scoliosis or other deformities. The palms, fingers, and toes appeared normal. The bilateral tendon reflexes were intact, and both the Trousseau and Chvostek signs were negative. Further evaluation was performed, and electrocardiography and ultrasonography of the kidneys, ureters, and bladder showed no abnormalities. Cranial CT revealed extensive, approximately symmetric calcified patches and linear foci in the bilateral cerebellar hemispheres, thalami, basal ganglia, and corticomedullary junctions of the cerebral hemispheres (**Figure 1**). Genetic testing identified a heterozygous mutation in the *CASR* gene in the patient, specifically c.2452T>C (p.Trp818Arg). The patient’s son also carried this mutation, while his wife was wild type (**Figure 1**). Although this mutation has not been previously reported, it was classified as likely pathogenic according to ACMG criteria (9) and supported by three prediction tools (PROVEAN, PolyPhen-2, and MutationTaster). Additional laboratory test results are summarized in **Table 1**.

The patient and his son were diagnosed as ADH1 in which the substitution of Trp with Arg at position 818 resulted in functional activation of *CASR*. The patient was started on oral calcium carbonate (0.6 g three times daily of elemental calcium), which was crushed and administered between meals, along with additional dietary calcium intake from 600 mL of milk per day (approximately 600 mg of elemental calcium), divided into three servings and taken separately from the calcium tablets. Following consensus-based recommendations for HP (10), Calcitriol (0.25 μg four times daily) was administered with meals, with each dose separated from calcium carbonate by several hours. The patient’s serum calcium level increased to 2.13 mmol per liter after 96h of supplementation, and 24-h urinary calcium excretion remained within the reference range. No further convulsive episodes occurred, and no additional therapies were required. Notably, the patient’s son, who was also treated with calcium supplementation and calcitriol, had no recurrence of symptoms or hypocalcemia.

### Cellular functional studies results

**Figure 2:**
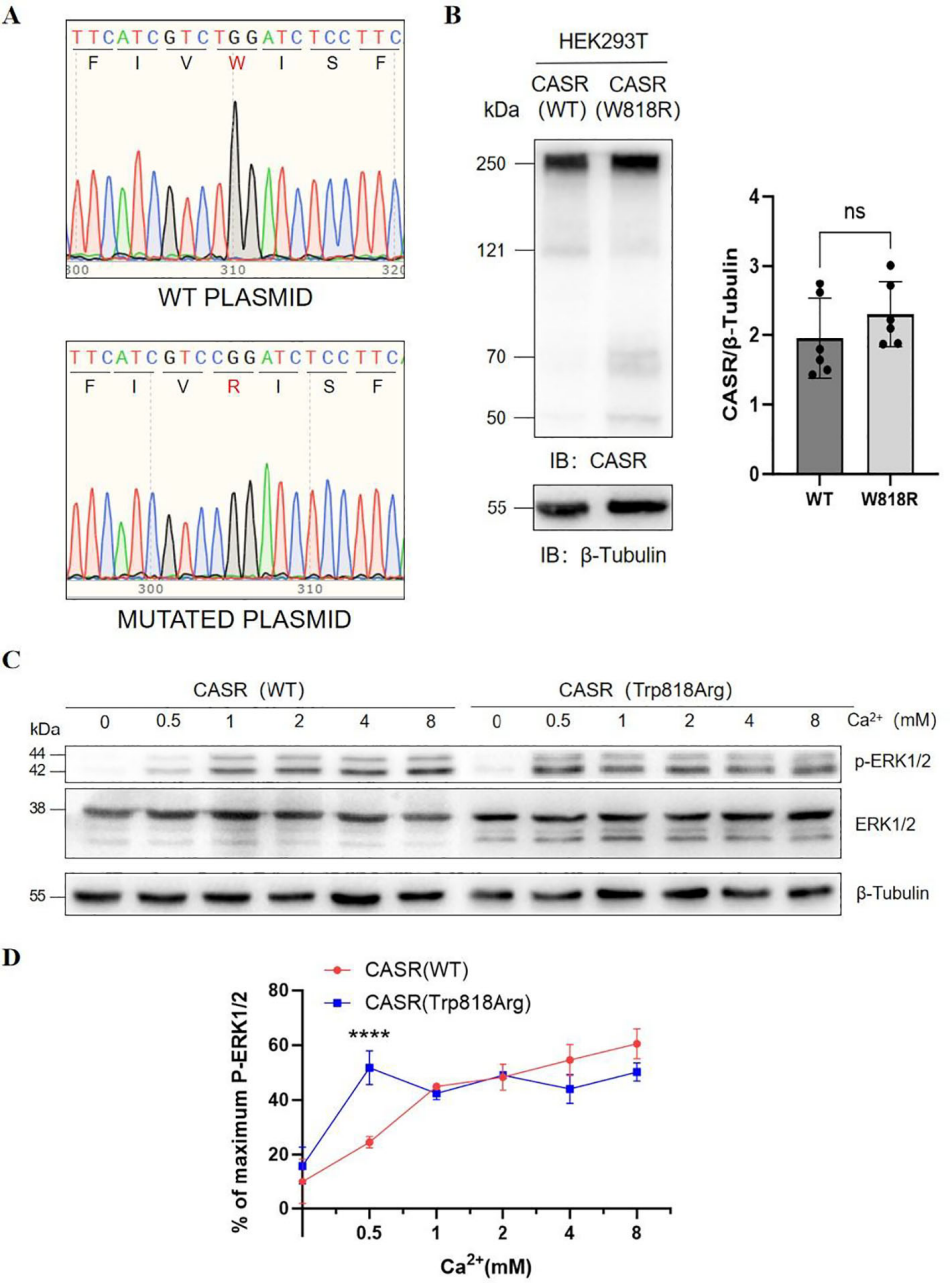
**(A)** Chromatograms of wild-type and mutant plasmid obtained by site-specific mutagenesis. **(B)** Western blot analysis of cell extracts from HEK293 cells transiently transfected with wild-type or W818R CASR. Cumulative data of CASR expression, normalized to β-tubulin, from 6 independent biological experiments are shown in the graph. The x axis indicates wildtype CASR protein and the W818R mutant species, and the y axis shows the relative CASR protein expression level. Values are plotted as the mean ± SEM. P values indicate an unpaired t-test carried out between wild-type and W818R CASR(n=6). **(C)** Ca^2+^-dependent activation of ERK1/2 in HEK293 cells transiently transfected with wild-type or W818R CASR. After transfection, cells were exposed to culture medium containing 0, 0.5, 1, 2, 4, or 8 mM Ca^2+^ for 30 min, followed by cell lysis and protein extraction. Levels of phosphorylated ERK1/2 (p-ERK1/2) and total ERK1/2 were assessed by western blot analysis, with band intensities normalized to β-tubulin. **(D)** Ca^2+^-dependent activation of ERK1/2 in HEK293 cells transiently transfected with wild-type or W818R CASR. The x-axis indicates the Ca^2+^ concentration, and the y-axis represents the ratio of phosphorylated ERK1/2 (p-ERK1/2) to total ERK1/2 (p-ERK1/2 plus ERK1/2). Data are expressed as a percentage of the mean value at each concentration. The two curves were then compared (values are shown as the mean ± SEM; n=3). Two-way ANOVA followed by *post-hoc* multiple comparisons test was used to compare the functional response of wild-type and W818R CASR across different conditions. The **** P value indicates a highly significant difference(adjusted P < 0.0001), while all other comparisons showed no significant difference (adjusted P > 0.05).

To assess the functional consequences of the identified mutation, wild-type and W818R mutant *CASR* expression constructs were generated and confirmed by Sanger sequencing to contain the c.2452T>C (p.Trp818Arg) substitution (**Figure 2A**). Using an *in vitro* cellular model, The western blot analysis demonstrated that the protein expression level of the W818R mutant was not significantly different from that of the wild-type receptor (**Figure 2B**). Second, the W818R variant exhibited enhanced ERK phosphorylation status activation compared to the wild-type receptor (**Figure 2C**). Subsequently, we assessed receptor activation in response to changes in extracellular calcium levels. Notably, when the cells were cultured under varying extracellular calcium conditions, we observed that the W818R mutation increased the sensitivity of the CASR-mediated MAPK pathway response to extracellular calcium (**Figure 2D**). These results confirmed that the substitution of Trp with Arg at position 818 resulted in functional activation of CASR.

## Discussion

The patient had presented with convulsions since birth and was treated with antiepileptic medications for several decades. However, convulsive episodes continued to occur intermittently. After admission, the constellation of severe hypocalcemia, hyperphosphatemia, low PTH levels, and intracranial multifocal calcifications prompted consideration of HP. When hypocalcemia occurs in the neonatal period, it can be classified according to the time of onset as early-onset or late-onset neonatal hypocalcemia. Early-onset neonatal hypocalcemia, which typically presents within the first 72 hours of life, generally requires calcium supplementation for at least 72 hours, whereas late-onset neonatal hypocalcemia usually presents after 7 days of life and often necessitates long-term therapy. When hypocalcemia is accompanied by hyperphosphatemia in the setting of normal renal function, HP should be strongly suspected (11). A positive family history should raise suspicion for a hereditary disorder and prompt genetic testing (12), which is important for diagnosis and cause identification. In our case, genetic analysis identified a previously unrecognized heterozygous mutation, c.2452T>C (p.Trp818Arg), in the *CASR* gene, and functional cellular studies demonstrated that this mutant results in activation of the CASR.

A previously reported *CASR* mutation affecting the same amino acid, c.2453G>T (p.Trp818Leu) (13), shares several features with our case. From a clinical perspective, both patients presented in the neonatal period with carpopedal spasm and seizure-like episodes. Biochemically, the degree of hypocalcemia was comparable, with serum calcium levels markedly below the normal range. Notably, vertical transmission to an offspring of the same sex was observed in both cases, and genetic testing confirmed heterozygous mutations. Although functional studies were not performed in previous case, our cellular analyses demonstrated that the c.2452T>C (p.Trp818Arg) mutation exhibits increased sensitivity to lower extracellular Ca^2+^ concentrations, representing a gain-of-function mutation, thereby contributing to the clinical manifestations of hypocalcemia.

CASR belongs to the class C G protein-coupled receptor family and functions as a homodimer on the cell membrane, with its subunits linked by disulfide bonds. It consists of a large N-terminal extracellular domain (ECD), a cysteine-rich module and a separate heptahelical transmembrane domain (14). Using high-resolution structural data, studies have revealed that more than one-third of the gain-of-function missense mutations associated with ADH1 and HP are localized at the homodimer interface of the ligand-bound, active CASR structure (15). CASR activation is achieved through rearrangements of the intracellular loop 2 and C-terminus, along with the involvement of natural polyamines and the amino acid L-tryptophan, which enhance receptor activation and allosteric modulation (14). Activating variants are typically clustered in two distinct regions: the extracellular domain of the receptor and the transmembrane helices 6 and 7 (16). These findings further suggest that amino acid changes in the 6th transmembrane region can lead to the activation of CASR, and the amino acid change in our patient is located precisely in the 6th transmembrane region.

CASR regulates extracellular calcium homeostasis via phosphorylation of ERK1/2 (17). Phosphorylation of ERK1/2 has been widely used to assess the responsiveness of mutant CASR to extracellular calcium compared with wild-type CASR (16, 18). Inherited or *de novo* activating variants of the CASR alter the set point for extracellular calcium, such that lower circulating Ca^2+^ levels are perceived as normal (5). In addition, gain-of-function mutations lower the activation threshold for calcium and cause a leftward shift in the calcium–response curve compared with the wild-type receptors (14, 19), indicating that activating mutants exhibit increased sensitivity to low extracellular calcium. In our study, the W818R variant exhibited significantly higher ERK1/2 phosphorylation at 0.5 mM calcium, suggesting enhanced sensitivity to low calcium. At higher concentrations (1.0–8.0 mmol/L), no difference was observed between the mutant and the wild-type. We speculate that receptor overactivation has reached a plateau, suggesting that activation may no longer occur or that activation is attenuated. However, relying on a single signaling pathway has limitations and may not fully reflect the activation status of the CASR. Future studies should incorporate additional downstream signaling pathways to more comprehensively elucidate the complex molecular mechanisms underlying the W818R variant.

Once the diagnosis of ADH1 is established, treatment options for symptomatic patients include calcium supplementation and calcitriol. However, to minimize the risk of hypercalciuria and nephrolithiasis, the target serum calcium concentration should be maintained between 1.8 and 2.1 mmol/L, at the lower end of the reference range (10). Given that patients with ADH1 often exhibit low or undetectable PTH, PTH-dependent renal 1-alpha-hydroxylase activity-responsible for conversion of 25-hydroxyvitamin D_3_ to its active form-is impaired. Consequently, active vitamin D analogs such as calcitriol or alfacalcidol are preferred in the management of ADH1, with a target serum vitamin D concentration of 20 ng/mL considered sufficient. A daily oral elemental calcium dose of 800–2000 mg is recommended for calcium supplementation (5, 6), and it was given in divided doses because single doses exceeding 500 mg have reduced intestinal absorption (20). In terms of diet, sodium transporters in the renal tubules reabsorb calcium from the ultrafiltrate, independent of glomerular filtration (21).Therefore, many guidelines recommend a low-sodium diet for patients with hypercalciuria (22–24).

Conventional treatments can raise serum calcium levels but do not address the underlying pathophysiologic mechanisms of ADH1. There are new therapeutic approaches specifically targeting ADH1. Among these, PTH analogs have shown significant promise, with PTH1-34, a bioactive N-terminal fragment of the PTH peptide, being the most extensively studied. In a study involving patients with ADH1 and nutritional rickets, compared to a once-daily regimen, a twice-daily regimen of PTH1–34 resulted in higher average serum calcium levels, without a significant increase in urinary calcium excretion (25). Another study on 12 children with severe congenital hypocalcemia, including seven ADH1, showed that PTH1–34 delivered via a pump required significantly lower doses compared to twice-daily subcutaneous injections. This approach normalized average serum calcium and magnesium levels, as well as urinary calcium excretion, while markedly decreasing bone turnover markers (26). More recently, a study involving six ADH1 patients who continued to experience hypocalcemic seizures despite conventional calcium and vitamin D analog therapy examined the effects of continuous subcutaneous infusion of PTH1–34 via a pump. After treatment, serum calcium levels increased, serum phosphorus levels decreased, and there was no worsening of renal calcification or increased calcium excretion. Additionally, both the frequency of seizures and hospitalizations significantly decreased (27).

In addition, negative allosteric modulators, or calcilytics, have emerged as a promising targeted therapy for ADH1. This class of drugs is divided into two main categories: amino alcohols and quinazolinones. Among the amino alcohols, NPSP795 demonstrated concentration-dependent increases in plasma PTH levels, up to 129% above baseline (p = 0.013), in five adults with ADH1 due to four distinct *CASR* mutations. Although fractional excretion of calcium decreased slightly, ionized calcium levels remained stable during treatment, suggesting effective regulation without calcitriol or substantial calcium supplementation (28). Additionally, a phase 2b study of Encaleret showed that twice-daily dosing, adjusted to achieve normal albumin-corrected calcium levels, corrected hypocalcemia and reduced hypercalciuria during both inpatient and 24-week outpatient periods. Treatment was associated with increased intact PTH, magnesium, and 1,25-dihydroxyvitamin D levels, while phosphorus and tubular phosphate reabsorption decrease. And encaleret was well-tolerated, with only mild, transient, and asymptomatic hypophosphatemia or hypercalcemia reported as treatment-related adverse events (29). For quinazolinones, compounds like ATF936 and AXT914, which bind irreversibly to the CASR transmembrane domain (30), demonstrated prolonged PTH release and improved calcium regulation in mice with gain-of-function *CASR* mutations (31). Overall, calcilytics represent a promising therapeutic avenue for ADH1, with potential to normalize calcium homeostasis while minimizing adverse effects.

## Conclusion

In conclusion, hypocalcemia should be considered in the evaluation of epilepsy in children. In cases of neonatal hypocalcemia accompanied by hyperphosphatemia and normal renal function, HP should be suspected. Genetic testing is essential for establishing a hereditary etiology of HP. In this study, we identified a novel heterozygous gain-of-function *CASR* mutant, c.2452T>C (p.Trp818Arg), expanding the mutational spectrum of ADH1.

## Data Availability

The original contributions presented in the study are included in the article/supplementary material, further inquiries can be directed to the corresponding author/s.
